# Developing a valid and reliable assessment of knowledge translation (KT) for continuing professional development program of health professionals

**DOI:** 10.7717/peerj.5323

**Published:** 2018-08-13

**Authors:** Irvin L. Ong, Michael Joseph S. Diño, Maria Minerva P. Calimag, Fe A. Hidalgo

**Affiliations:** 1 Research Development and Innovation Center, Our Lady of Fatima University, Valenzuela City, Philippines; 2 The Graduate School, University of Santo Tomas, Manila, Philippines; 3 Phi Gamma Chapter, Sigma Theta Tau International Honor Society of Nursing, Indianapolis, IN, United States of America

**Keywords:** Knowledge translation, Continuing professional development, Health professionals, Learning assessment, Tool development, Validity testing, Reliability testing, Vignette, Reflective assessment, CPD

## Abstract

**Introduction:**

Knowledge Translation (KT) is expected to be a critical learning outcome of a Continuing Professional Development (CPD) program. It continues to serve as an area of interest among educators and healthcare providers due to its importance to evidence-based practice. This study endeavored to develop a valid and reliable KT learning assessment tool in CPD.

**Methods:**

The Inventory of Reflective Vignettes (IRV), an innovative approach of integrating research vignettes, was utilized in crafting the 20-item IRV-KT tool. This instrument includes knowledge creation and action as essential KT constructs. KT competency was assessed in three segments (i.e., before and after CPD event and if in a lecture) using a one-group post-posttest pre-experimental design. Health professionals who successfully completed a CPD program on a knowledge translation topic were asked to complete the IRV-KT during the pilot study (*n* = 10) and actual implementation (*n* = 45). Responses were subjected to Cronbach’s reliability and criterion-validity testing.

**Results:**

The initial test of the IRV-KT tool demonstrated a high internal reliability (*α* = 0.97) and most items yielded acceptable validity scores. During the actual implementation, a higher reliability score of 0.98 was generated with significant correlations between the before-after segments for both KT constructs of creation (*r* = 0.33, *p* < 0.05) and action (*r* = 0.49, *p* < 0.05). All items have significant positive validity coefficients (*r* > 0.35, *p* < 0.05) in all segments of the tool.

**Discussion:**

The study produced a reflective assessment tool to validly and reliably assess KT learning in a CPD. IRV-KT is seen to guide the curriculum process of CPD programs to bridge learning and healthcare outcomes.

## Introduction

Knowledge Translation (KT) has been prominent in the health fields (Lang, Wyer & Haynes, 2007; Lencucha, Kothari & Rouse, 2007; Armstrong et al., 2013), and its primary focus has been mostly on practice and research, with some focus on educational and professional development (Urquhart et al., 2013). As a learning approach, KT has no agreed upon conceptual framework and platform (Pablos-Mendez & Shademani, 2006). However, KT seems to have a resemblance to (a) constructivist learning theory (Thomas et al., 2014) on the premise that learners construct new knowledge, as well as (b) outcomes-based education on transforming the declarative knowledge into functioning knowledge in a professional education (Biggs & Tang, 2007; Biggs & Tang, 2011). These principles put forward the importance of integrating knowledge translation in any teaching and learning activity such as in Continuing Professional Development (CPD). There are limited embedded assessments that would capture its natural occurrence in various learning contexts, more so in a CPD environment. Thus, this study worked toward the development of a valid and reliable assessment tool to use in measuring KT competency as a CPD learning outcome. For instance, nurse managers who aspire to conduct KT CPDs as part of their in-service program shall benefit from the tool, as well as academicians who need a valid and reliable instrument to measure trainees’ achievement of KT training outcomes.

### Foundation of knowledge translation (KT)

The universal “know-do” gap (i.e., the difference and gap between theory, knowledge, evidence or research, and practice, application, policy or decision) remains a critical challenge even in the contemporary world (Pablos-Mendez & Shademani, 2006; Santesso & Tugwell, 2006; Mustard, 2010; Grimshaw et al., 2012; Atchan, Davis & Foureur, 2014; Health Policy Project, 2014; Zhang et al., 2015). Although knowledge possesses an inherent catalytic potential in advancing the globalized society such as inclusive knowledge societies and knowledge-based economy (CEDEFOP, 2014; Pablos-Mendez & Shademani, 2006; Souter, 2014; UNESCO-IBE, 2013), it requires meaningful and contextual applications to elicit desired outcomes (Santesso & Tugwell, 2006; Bassi et al., 2013). Both CPD and KT offer mutually reinforcing strategies (Graham et al., 2006; Davis & Davis, 2010; Kitto et al., 2013) in placing knowledge to work. In essence, this stresses three-sided (i.e., researchers, educators and practitioners) collaboration (Bjørk et al., 2013) that is similar to the European Commission’s (2015) knowledge triangle—education, research, and innovations.

Several authors (Ho et al., 2004; Wallin, 2009; Metzler & Metz, 2010; Kothari & Armstrong, 2011; Straus, Tetroe & Graham, 2011; Sibley et al., 2011; Collisson et al., 2011; Kastner & Straus, 2012; Muntaner et al., 2012; Wolfe et al., 2012; Bassi et al., 2013; Gholami et al., 2013; Urquhart et al., 2013; Bjørk et al., 2013) have espoused the widely accepted Canadian Institutes of Health Research’s (2014) definition of KT as a continuous knowledge processing (i.e., synthesis, dissemination, exchange and application) within a complex interactive system of researchers and knowledge users. Correspondingly, knowledge-to-action (KTA) process (Graham et al., 2006) generally illustrates KT that offers an alternative framework to bridge the know-do gap in the 21st century. It emphasizes the cyclic coexistence of knowledge creation and application. During the creation phase, knowledge needs to undergo a refinement process such as inquiry, synthesis and development. Whereas, the action part involves activities needed for actual knowledge application such as adaptation, intervention, evaluation, and continuation.

Graham et al. (2006) clarified commonly shared concepts of KT. *Knowledge transfer* exclusively describes a linear top-down approach (Visram, Goodall & Steven, 2014), and one-way (or impliedly can be two-way) process of getting knowledge with no actual application. This concept reflects a mere sending of information to a user (Majdzadeh et al., 2008), though Froese & Montgomery (2014) have viewed knowledge transfer synonymously with knowledge translation. These views concur with Choi & Johanson’s (2012) comparison of the (a) *static*, (b) *dynamic* and (c) *knowledge translation perspectives* of knowledge transfer, respectively. In the same way, *knowledge exchange* as a two-way process (Visram, Goodall & Steven, 2014) has been conceived to release some restrictions of transfer. Knowledge exchange expands the transfer concept by emphasizing the collaborative roles between the researchers, leaders, and stakeholders. Though this concept expansion, relevant knowledge is available, although involvement may vary throughout the process. Several studies have compounded these terms as *knowledge translation and exchange (KTE)* to further strengthen the involved interactive mechanisms (Légaré et al., 2011; Wolfe et al., 2012; Visram, Goodall & Steven, 2014). On the contrary, * research utilization* (or implementation) directs its effort on the sole use of research evidences. This concept shares almost a similar stand with *evidence-based practice (EBP)* that uses the best evidence as the ideal knowledge in guiding decision and practice (Wallin, 2009; Metzler & Metz, 2010; Atchan, Davis & Foureur, 2014). Lastly, *translational research* may seem similar, yet it deals with the transfer of basic scientific knowledge to clinical application. In contrast, Grimshaw et al. (2012) embraced the view of translation research as their frames for knowledge translation. This view refers to the second type of translational research, which deals with improving health outcomes using new clinical knowledge. In spite of their differences, these concepts are subsumed under the broader context of KT.

### Strategies of Knowledge Translation (KT)

Despite availability of many theoretical supports and models (Sibley et al., 2011; Brehaut & Eva, 2012; Larrivée, Hamelin-Brabant & Lessard, 2012; Thomas et al., 2014), there has been no single strategy for KT (Metzler & Metz, 2010; Gholami et al., 2011; Grimshaw et al., 2012; Kastner & Straus, 2012; Visram, Goodall & Steven, 2014). At large, the Canadian Institute of Health Research (Canadian Institutes of Health Research, 2014) has specified two (2) general KT approaches. The traditional (Kothari & Wathen, 2013) *end-of-grant knowledge translation* empowers the researcher to make the stakeholders (e.g., users and clients) and their peers aware of the research evidence as knowledge. This translation may involve typical research dissemination (e.g., conferences, presentations, and publications) and intensive activities such as interactive sessions, educational events and knowledge brokering. As for the *integrated knowledge translation (IKT)*, the knowledge researcher and user engage in a meaningful collaboration toward sustainable outcomes. Its strategies may include action research, knowledge co-creation and participatory methods (Tetroe, 2007). Likewise, Collisson et al. (2011) have pointed out that these KT approaches share a direct link with their knowledge production mode. The independent knowledge production leads to the academic merits of end-of-grant KT, while collaborative generation facilitates integrated KT for a direct contextual application.

Another KT strategy integrates modern technology to yield a *Technology-Enabled Knowledge Translation (TEKT)* (Bassi et al., 2013; Urquhart et al., 2013). This strategy embodies the convergence of technology and innovation with KT, which supports information collection, production and distribution (Pablos-Mendez & Shademani, 2006; Majdzadeh et al., 2008; Urquhart et al., 2013). Several scholars (Ho et al., 2004; Bassi et al., 2013) have maintained positive views on TEKT’s potentials for online education, web-based intervention, and integrated systems. Similarly, *informatics-based knowledge translation* optimizes the utilization of information systems for adjunctive mechanisms such as clinical decision support systems and healthcare information systems (Kastner & Straus, 2012). Relatedly, social media open up new opportunities in reinforcing knowledge translation activities and interactions (Hamm et al., 2013). In other situations, the social environment becomes the strategic center of knowledge translation such as the community of practice (Lencucha, Kothari & Rouse, 2007; Urquhart et al., 2013), virtual community (Bassi et al., 2013) and community-based settings (Kothari & Armstrong, 2011).

Interestingly, most KT strategies tend to hold educational activities (Zwarenstein & Reeves, 2006; Majdzadeh et al., 2008; Wallin, 2009; Reitmanova, 2009; Davis & Davis, 2010; Straus et al., 2011; Wahabi & Al-Ansary, 2011; Grimshaw et al., 2012; Larrivée, Hamelin-Brabant & Lessard, 2012; Armstrong et al., 2013; Bassi et al., 2013; Thomas et al., 2014). Regardless of whether a standalone or combined intervention is used, KT activities may include learning activities (e.g., seminar, lectures, workshops, outreach, trainings and meetings) (Graham et al., 2006; Wahabi & Al-Ansary, 2011; Grimshaw et al., 2012; Armstrong et al., 2013; Bassi et al., 2013; Urquhart et al., 2013) and CPD (Grimshaw et al., 2006; Pablos-Mendez & Shademani, 2006; Santesso & Tugwell, 2006; Davis & Davis, 2010; Metzler & Metz, 2010). Under these circumstances, KT and CPD can be overlapping and confusing (Graham et al., 2006). Kitto et al. (2013) expounded the harmonies, distinctions, and associations of these academic domains. Both KT and CPD aspire to improve collaborations, services, and outcomes, though their underlying approach may differ. CPD gives attention to developing competence and performance of health professionals (Enriquez et al., 2015), whereas KT ensures quality information and tools for the stakeholders. Despite their differences, KT and CPD maintain a mutual relationship and share common challenges. KT adapts theoretical foundations from CPD, while CPD exploits KT as a strong rationale for its strategies.

## Subjects and Methods

In pursuit of developing a reflective tool for assessing KT learning in a CPD program, this study embarked on utilizing the tool development framework of IL Ong, MJS Diño, MMP Calimag & FA Hidalgo (2017, unpublished data). In their work, they developed the Inventory of Reflective Vignettes (IRV) to guide them in designing a valid and reliable tool for interprofessional learning. Reflective tests utilize vignettes or hypothetical scenarios for reflection (Datta Gupta, Kristensen & Pozzoli, 2010; Hudson & Cairns, 2014; Gesinde, Temitope & David, 2014) that are proven to be highly correlated with the actual outcomes (Colón-Emeric et al., 2018). It can propel concrete thinking and elicit opinions (Santos-Eggimann & Meylan, 2017). The IRV integrated a six-point Likert scale for reflective measurement of desired competencies (a) prior CPD, (b) upon CPD completion, and (c) during a vignette situation. Following the tool development process, the content design involves literature reviews (Shrader et al., 2017) in identifying key constructs of KT and corresponding items. The designed tool went through expert review (*n* = 5) for face validation. Similarly, a one-group post-posttest pre-experimental design was adopted in this study to establish the validity and reliability measures of the designed KT assessment tool. Post-posttest design refers to the assessment wherein the learners are tasked to reflect on their prior and current competency level (MacDonald et al., 2010). Implementation of this design avoids bias by eliminating pre-assessment survey, but pre-assessment items were included as part of the post-assessment survey (Bottenberg et al., 2013). In addition, the IRV-KT tool primarily intends to provide a self-assessment of KT competency through reflective learning. The study focused on establishing tool validity and reliability for its potential use in any CPD program. As for the assessment of the learning outcomes, the CPD program had its own authentic performance tests with corresponding scoring rubrics.

After the research protocol was approved by an accredited ethics review committee of the Philippine Health Research Ethics Board, the pilot study (*n* = 10) was conducted on November 2017 in a university-offered CPD program using the exploratory method of teaching for health professionals. In this study, health professionals refer to licensed physicians, nurses and allied health workers like physical therapists and pharmacists. All respondents gave written consent prior the CPD program and their privacy was maintained throughout the study. The pilot sample consisted of nurses pursuing postgraduate degrees with equal distribution in terms of gender. The initial Cronbach’s analysis (Table 1) underscores the high internal reliability (*α* = 0.97) of the designed tool. IRV-KT has excellent item consistency in estimating KT learning in each segment that is before (*α* = 0.96), after (*α* = 0.96), and if (*α* = 0.99). This is likewise reflected on the identified KT constructs, which are creation (*α* = 0.96) and action (*α* = 0.96).

**Table 1:** Pilot test reliability (*n* = 10) of the IRV-KT tool.

Constructs (*n* of items)	Before	After	If	Overall
Creation (*n* = 10)	0.95	0.95	0.99	0.96
Action (*n* = 10)	0.94	0.93	0.99	0.95
Overall	0.96	0.96	0.99	0.97

The preliminary analysis also tested the IRV-KT for validity. Its initial face and content validation provided the tool with a criterion. To estimate the validity coefficient, each item was correlated with the summative score of all segments. The statistical validity (Table 2) presented varying coefficients. All “If” (*n* = 20) and many “Before” (*n* = 14) items yielded significant correlations (*p* < 0.05), whereas most “After” items failed to demonstrate criterion validity.

**Table 2:** Pilot validity testing (*n* = 10) of the IRV-KT tool.

KT constructs and items	Segments (r)		
	Before	After	If
Creation			
*Share my thoughts/ideas*	0.53	0.52	0.70^*^
*Retrieve relevant information*	0.90^*^	0.19	0.96^*^
*Explain the need for information*	0.81^*^	0.61	0.87^*^
*Analyze the usefulness of information*	0.69^*^	0.48	0.86^*^
*Evaluate the information*	0.83^*^	0.69^*^	0.86^*^
*Appreciate literature reviews*	0.73^*^	0.28	0.91^*^
*Determine the gaps/needs*	0.59	0.69^*^	0.87^*^
*Recommend useful solution*	0.93^*^	0.83^*^	0.90^*^
*Work well with others*	0.52	0.03	0.78^*^
*Develop new products*	0.72^*^	0.32	0.85^*^
Action			
*Apply a team approach*	0.84^*^	0.22	0.81^*^
*Use knowledge in many situations*	0.59	0.34	0.89^*^
*Fit my knowledge within a context*	0.63^*^	0.32	0.84^*^
*Apply my knowledge*	0.51	0.58	0.81^*^
*Build my confidence*	0.34	−0.20	0.90^*^
*Make sound decisions*	0.69^*^	0.14	0.84^*^
*Recognize the knowledge in action*	0.69^*^	0.48	0.79^*^
*Reflect on my knowledge use*	0.76^*^	0.76^*^	0.79^*^
*Enjoy getting feedback*	0.65^*^	0.00	0.75^*^
*Share the results with others*	0.73^*^	0.01	0.81^*^

**Notes.**

* Significant at 0.05 alpha level.

IRV-KT went through further improvement using the findings from the pilot testing and focus group discussion. A small sample pilot study was intended to supply preliminary evidence of the tool applicability of use (Kennedy et al., 2018). The statements of each item were reworded for clarity and specificity. In the actual implementation on December 2017, the study participants (*n* = 45) were tasked to accomplish the IRV-KT at the end of the non-lecture CPD program for health professionals. Majority of the actual respondents are female (60%) nurses (36%) with master’s degrees (40%) or units (22%). Their responses were analyzed to determine its test reliability (Batterham & George, 2003) and criterion-related validity (Peeters & Martin, 2017).

## Results

The study evaluated the designed the IRV-KT tool (Tool S1) for validity and reliability of its measures. After the actual implementation (*n* = 45), the IRV-KT maintained its excellent internal reliability (Table 3) with Cronbach’s coefficient of 0.98 which is higher than the pilot result. Its high internal consistency was cascaded down to all constructs (*α* = 0.97) of every segments (*α* = 0.98). This result indicates that IRV-KT can generate a reliable measure of KT learning competencies.

**Table 3:** IRV-KT test reliability (*n* = 45).

Constructs (*n* of items)	Before	After	If	Overall
Creation (*n* = 10)	0.98	0.97	0.96	0.97
Action (*n* = 10)	0.96	0.97	0.97	0.97
Overall	0.98	0.98	0.98	0.98

The correlation study regarding segments (Table 4) shows the presence of significant relationship among segments. Surprisingly, only the before and after segments have statistical significance in the creation (*r* = 0.33, *p* < 0.05) and action (*r* = 0.49, *p* < 0.05) constructs. Although this is expected, the before and after segments have a limited relationship. This finding suggests that IRV-KT can provide reliable and, at the same time, relative estimates of KT learning competency in actual (i.e., before and after CPD) and hypothetical (i.e., vignette) situations.

**Table 4:** Relation matrix of KT constructs as to segments.

Constructs	Segments (r)		
	Before	After	If
Creation			
Before	–		
After	0.33^*^	–	
If	0.28	0.01	–
Action			
Before	–		
After	0.49^*^	–	
If	0.16	0.19	–

**Notes.**

* Significant at 0.05 alpha level.

The estimates of statistical validity (Table 5) show the correlations of the items in every construct. All items have significant positive validity coefficients (*r* > 0.35, *p* < 0.05) in all segments. This outcome strongly implies that the IRV-KT is considered to be a beneficial tool in assessing KT learning competencies at different segments. Moreover, it was able to measure baseline performance indicating postdictive validity and vicarious experience showing predictive validity. Thus, the IRV-KT can assess KT as a learning outcome of both actual and circumstantial CPD.

**Table 5:** Criterion-related validity measures of IRV-KT.

KT constructs and items	Segments (r)		
	Before	After	If
Creation			
*Share my thoughts/ideas with others*	0.64^*^	0.47^*^	0.52^*^
*Retrieve relevant evidences/information*	0.71^*^	0.52^*^	0.56^*^
*Explain the need for searching scientific information*	0.72^*^	0.61^*^	0.57^*^
*Analyze the usefulness of a reliable information*	0.76^*^	0.51^*^	0.55^*^
*Evaluate the data and information critically*	0.73^*^	0.58^*^	0.55^*^
*Appreciate the value of reviewing literatures/sources*	0.66^*^	0.55^*^	0.50^*^
*Determine the gaps/needs of a given situation*	0.68^*^	0.53^*^	0.63^*^
*Recommend useful solution/information for application*	0.68^*^	0.59^*^	0.58^*^
*Work well with others in making plans/strategies*	0.64^*^	0.55^*^	0.51^*^
*Develop new knowledge/products based on the needs*	0.71^*^	0.59^*^	0.65^*^
Action			
*Apply a team approach in the use of knowledge*	0.43^*^	0.61^*^	0.44^*^
*Use previous and current knowledge in many situations*	0.63^*^	0.66^*^	0.71^*^
*Fit my knowledge within a specific context/situation*	0.60^*^	0.69^*^	0.72^*^
*Apply my knowledge to individual situation*	0.64^*^	0.72^*^	0.68^*^
*Build my confidence in using my knowledge*	0.68^*^	0.65^*^	0.66^*^
*Make sound decisions using my knowledge*	0.64^*^	0.68^*^	0.64^*^
*Recognize the value of using knowledge in real action*	0.61^*^	0.63^*^	0.59^*^
*Reflect on my actual use of knowledge in a situation*	0.63^*^	0.74^*^	0.68^*^
*Enjoy getting feedback on how I use my knowledge*	0.47^*^	0.62^*^	0.61^*^
*Share the results of my knowledge use with others*	0.49^*^	0.68^*^	0.46^*^

**Notes.**

* Significant at 0.05 alpha level.

## Discussion

### IRV-KT as a valid and reliable tool

The study findings confirm the validity of the IRV-KT tool. As expected, this instrument would provide valid measures of KT learning, because it was developed using substantial sources. Its strength as a learning tool primarily rests on its capacity to offer reproducible results in both technical (i.e., style and format) and instructional (i.e., content and learning process) aspects of KT as a desirable outcome of a CPD program. The IRV-KT is also reliable in assessing KT constructs of ‘creation’ and ‘action’ that resulted in commendable overall tool reliability.

KT, an area of interest in continuing professional education, is a constructive response to fill in the gray space within the “know-do gap” (Bero et al., 1998; Thompson et al., 2007). For instance, it has been reported that high-quality evidence is not found in actual professional practice (Majumdar, McAlister & Furberg, 2004; Larrivée, Hamelin-Brabant & Lessard, 2012; Negrini et al., 2018). In the field of education, the IRV-KT tool could serve as a potent instrument to ascertain KT competency outcomes among health professionals as lifelong learners in eliciting evidence-based policies, decisions, and practices.

### IRV-KT as a reflective and meaningful assessment

The IRV-KT tool is considered useful because it can cultivate a more profound reflection on self-performance and authentic functioning knowledge. Further, it provides a concrete mechanism for metacognitive thinking through awareness of one’s learning (Montagna, Benaglio & Zannini, 2010). Self-reflection has been proven as a valuable exercise in both learning (Semradova & Hubackova, 2015) and instruction (Williamson, 2018). In previous literatures, it is being used to evaluate teaching methods, strategies, and approaches (Klimova, 2014). Reflecting on one’s learning further improves critical thinking and insight that are directly translatable to actions and changes of behavior (Lew & Schmidt, 2011; Asselin & Fain, 2013; Gardner, 2013).

It is essential to assess KT among health professionals for it directly impacts practice quality (Pablos-Mendez & Shademani, 2006). The IRV-KT tool boasts its capacity to identify key processes of knowledge translation through its constructs. It focuses on specific, concrete tasks (and not purely theoretical dimensions) involved in epistemic construction and application. Thus, educators can be significantly benefitted from the direct utilization of this tool in the field of health sciences and healthcare, more so in the CPD learning context.

### IRV as a practical tool development framework

Like in the previous study (IL Ong, MJS Diño, MMP Calimag & FA Hidalgo, 2017, unpublished data), using the IRV framework and its procedures can produce valid and reliable reflective assessments. It provides a down to earth framework that is easy to incorporate in real-world scenarios. The demand for instruments focusing on Knowledge Translation is increasing (Ellen et al., 2017), but remains to be a gray area of literature (Chen et al., 2017). Whilst most are focused initially on validity and reliability concepts (Hinkin, Tracey & Enz, 1997), fewer studies have dwelt on the pragmatic educational usage of research instruments. In these cases, the expeditiousness of using the IRV framework is a valuable add-on in developing more assessment and research tools with practical value.

## Conclusion

The study designed the IRV-KT and established its validity and reliability in assessing KT in CPD. It identified creation and action as the fundamental constructs of KT. The findings also emphasized the educational value of IRV-KT in providing a reflective assessment of KT competencies and IRV framework in developing sound learning evaluations. This study afforded a solid strategy on KT assessment using post-post test administration. Educational leaders, planners, and implementers in the health sciences are encouraged to utilize the IRV-KT beyond CPD contexts in the evaluation of both teaching and learning. Cognizant of the study limitations and constraints, further studies need to explore IRV-KT by construct validity testing to determine its convergent and discriminant interrelationships. IRV-KT is consequently viewed to be an indispensable instrument for guiding the development, implementation, evaluation, and improvement of CPD programs for health professionals.

##  Supplemental Information

10.7717/peerj.5323/supp-1 Supplemental Information 1 Inventory of Reflective Vignettes—Knowledge Translation (IRV-KT) tool

10.7717/peerj.5323/supp-2 Supplemental Information 2 Raw data

## Additional Information and Declarations

### Data Availability

The following information was supplied regarding data availability: The raw data are provided in a Supplemental File.
