# Supplementary material for: Developing a valid and reliable assessment of knowledge translation (KT) for continuing professional development program of health professionals
Source: PeerJ. 2018 Aug 13;6:e5323. doi: 10.7717/peerj.5323 (PMC6095105; doi:10.7717/peerj.5323)
Supplement: Supplemental Information 1 [file peerj-06-5323-s001.pdf]

INVENTORY OF REFLECTIVE VIGNETTES – KNOWLEDGE TRANSLATION (IRV-KT)

INSTRUCTION
For each statements below, please rate your answer per column that describes your level of performance as follows:

1 = Emerging; 2 = Developing; 3 = Minimal; 4 = Proficient; 5 = Advanced; 6 = Excellent

IRV-KT	BEFORE the CPD program, I was able to:						AFTER the CPD program, I am able to:						IF I joined a LECTURE program, I'd be able to:					
**Creation**: *constructing knowledge individually and/or socially*	1	2	3	4	5	6	1	2	3	4	5	6	1	2	3	4	5	6
• Share my thoughts/ideas with others																		
• Retrieve relevant evidences/information																		
• Explain the need for searching scientific information																		
• Analyze the usefulness of a reliable information																		
• Evaluate the data and information critically																		
• Appreciate the value of reviewing literatures/sources																		
• Determine the gaps/needs of a given situation																		
• Recommend useful solution/information for application																		
• Work well with others in making plans/strategies																		
• Develop new knowledge/products based on the needs																		
**Action**: *putting knowledge to work in another situation*	1	2	3	4	5	6	1	2	3	4	5	6	1	2	3	4	5	6
• Apply a team approach in the use of knowledge																		
• Use previous and current knowledge in many situations																		
• Fit my knowledge within a specific context/situation																		
• Apply my knowledge to individual situation																		
• Build my confidence in using my knowledge																		
• Make sound decisions using my knowledge																		
• Recognize the value of using knowledge in real action																		
• Reflect on my actual use of knowledge in a situation																		
• Enjoy getting feedback on how I use my knowledge																		
• Share the results of my knowledge use with others																		
